# Improved emergency activation in interventional radiology reduces procedure delay and facilitates treatment success in patients with acute arterial bleedings

**DOI:** 10.1371/journal.pone.0313008

**Published:** 2024-11-12

**Authors:** Jonathan Nadjiri, Marc Mühlmann, Tobias Waggershauser, Tobias Geith, Philipp M. Paprottka

**Affiliations:** Department of Interventional Radiology, School of Medicine, University Hospital Klinikum Rechts der Isar, Technical University of Munich, München, Germany; Ascension Sacred Heart Hospital Pensacola, UNITED STATES OF AMERICA

## Abstract

**Background:**

Research of interventional treatment success in arterial bleeding cases is almost exclusively focused on technical and procedural factors. This study investigates the effect of an improved preprocedural activation algorithm for acute arterial bleedings treated by interventional radiology.

**Methods:**

During the three-year study period (2018–2021), the authors implemented an always-reachable, simple-to-remember emergency phone number routed to the responsible interventional radiologist on call and compared this pathway to the previous activation process. Data were acquired for all emergency cases with active arterial bleeding detected in CT scans and the diagnosis to treatment intervals before and after implementation were retrospectively analysed. Time signatures in CT and angiography were used to determine the interval.

**Results:**

1322 calls or contacts occurred during the study period. In general, 625 emergency procedures were conducted; 120 bleeding interventions met the study requirements. In the study 44 patients were treated via the conventional pathway and 76 via the emergency phone activation. The activation algorithm utilizing the emergency phone led to a slight decrease in radiation doses and fluoroscopy time and a significant reduction (15min) in diagnosis to treatment intervals (p = 0.019). After implementing the emergency phone, the technical success rate increased significantly from 68% to 94% (p<0.001).

**Conclusions:**

This study shows that effective communication structures, such as implementing a standardized activation pathway via an emergency phone, can significantly reduce diagnosis to treatment intervals and increase technical success rates. Effective communication is crucial for interventional radiology to deal with acute and life-threatening conditions requiring immediate treatment. This study presents a possible improvement and provides valuable insight for interventional radiology clinics seeking to optimize their communication and management strategies for emergency cases.

## Background

Communication and human errors are common sources of unfavourable clinical outcomes and management in emergency cases [1–6]. Acute arterial bleeding is associated with high mortality but can be treated effectively by interventional radiology utilizing embolization techniques [7].

**Fig 1:**
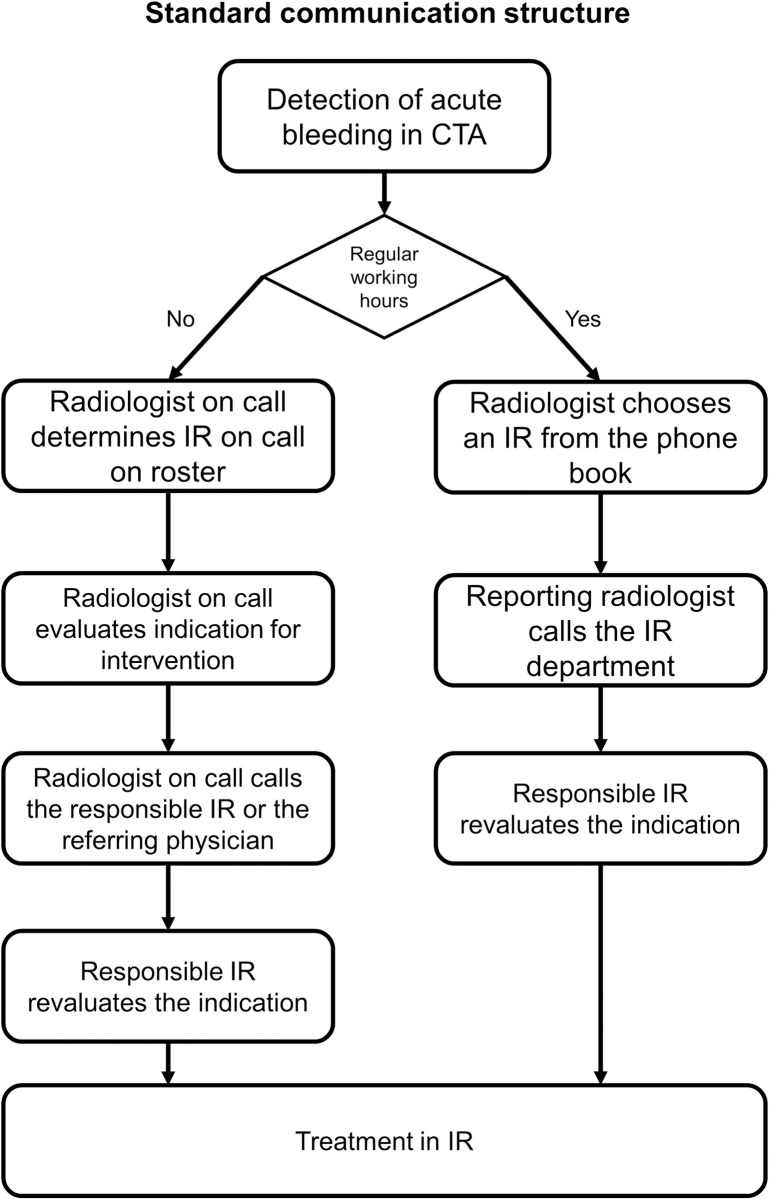
Illustrates the activation pathway of interventional radiology (IR) without an emergency phone in and outside regular working hours. This is an example of activation via the diagnostic radiologist. Other clinical partners would have to contact the diagnostic radiologist first outside the normal working hours to determine the IR on call.

Intra-clinical communication has been found to be key of successful for a long time [8]. Yet, publications about practical activation algorithms and suggestions about their implementation are rare. Is has been published that steamlining activation processes of interventional therapy is beneficial for the patients outcome [9]. Still communication structures in that publication rely on in clinic calls and parallel structures. Recent study about calling the interventional radiologist for emergency cases highlight the importance of early contact but also point out inefficient communication structures promoting even more pre-emptive calls [6]. Until August 2020, the in-house standard of IR (Interventional Radiology) activation in acute bleeding cases differed between working hours and night shifts. Outside of regular working hours, the diagnostic radiologist on call reviewed the CT scan, screened for possible treatment indications, and determined the responsible IR and the assisting personnel using a printed-out roster before calling each team member via their private mobile phone numbers. Additional phone calls between IR and the referring physician were frequently necessary to re-evaluate the treatment indication or to collect additional patient information. This concept was prone to ineffective communication because of its structure. Time delays occurred, for instance, when other emergencies occupied the attention of the diagnostic radiologist, but also whenever other involved physicians wanted to discuss the case at hand, as multiple individual phone numbers resulted in a laborious and time-consuming communication chain. During regular working hours, the reporting radiologist usually called the more generally known IR department numbers. As an outlined responsible IR was non-existent, activation of the treatment was uncoordinated, and calls could be addressed to the wrong contact persons resulting in several phone calls, thereby potentially hindering activation of the IR treatment. These activation methods are prone to causing treatment delay and misinformation. The initial activation pathway is shown in Fig 1.

Therefore, an emergency phone was established with an easy-to-remember four-number combination. This number and its purpose were distributed throughout the clinic and it is still part of every IR report. The phone itself is routed to the mobile phone of the responsible physician on call. Consequently, calls to this number can be answered directly 24 hours per day, seven days a week, and 365 days per year by an experienced IR.

This step led to a substantial increase in contact between referring and IR physicians.

**Fig 2:**
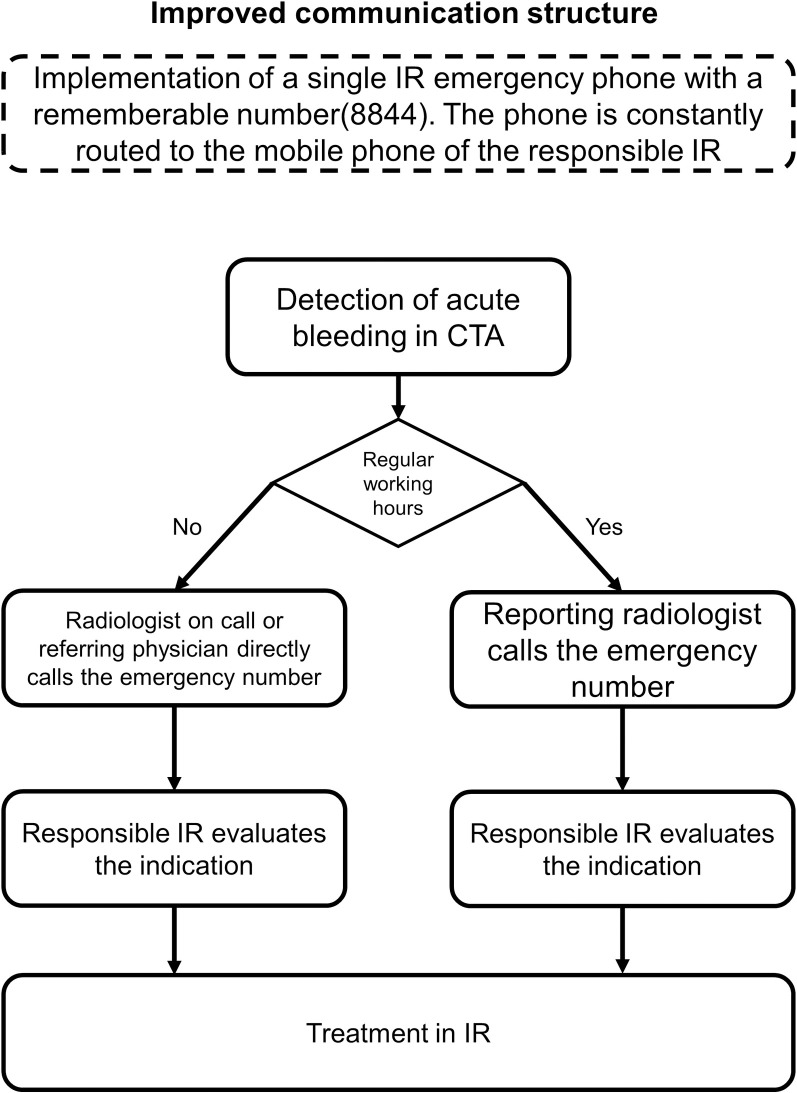
Illustrates the activation pathway of interventional radiology (IR) after implementing an emergency phone in and outside regular working hours. This is an example of activation via the diagnostic radiologist. Other clinical partners can contact the IR directly during and outside regular working hours.

Hereafter we investigate the effect of an improved activation algorithm for acute arterial bleedings treated by interventional radiology. We hypothesize that simplified structures allow for shorter intervals between diagnosis and treatment. The improved algorithm is shown in Fig 2.

## Methods

### Acquisition of data

All emergency cases between 2018 and 2021 with acute active arterial bleedings detected in CT were included. The time between the last CT image and the first angiography series was used to determine the diagnosis-treatment interval. This interval was used because time signatures in the images are the most accurately documented surrogats. Bleedings diagnosed with ultrasound, post-operative bleedings, and other emergencies were excluded as time intervals could not be retraced as accurately. Further, complex interventions such as emergency TIPSS and PTCDs were excluded because the indication process is more multifaceted and requires multidisciplinary discussions. Therefore, these intervals might not be suitable surrogates for evaluating the effectiveness of communication structures. The study interventions were conducted by all five senior interventional radiologists of the department. Years of experience in interventional radiology (fulltime interventional radiology) were 6, 9, 12, 23 and 39 years. Separate analyses of physicians was not performed as the team on call was identical to the period before and after introduction of the new activation pathway. Data was accessed for research purposes on 31/12/2020.

### Study population and parameters of interest

Parameters of interest were the type of intervention, gender, patient’s age, the origin of bleeding, dose, intervals, technical success, procedure times, and type of treatment.

### Statistics

To test for the study hypotheses, intervals from diagnosis to treatment were compared for patients before and after implementing the emergency phone utilizing a t-test. R Statistics (R version 3.5.3 (2019-03-11) — "Great Truth") was used for dedicated and descriptive statistics [10].

### Ethics approval and consent to participate

This study is approved by the local ethics committee (Ethikkommission der Fakultät für Medizin der Technischen Universität München). All procedures performed in studies involving human participants were in accordance with the ethical standards of the institutional and national research committee and with the 1964 Helsinki declaration and its later amendments or comparable ethical standards. This is a retrospective study of healing attempts; consent for participation was waived by the local ethics committee. Informed consent was waived by the ethics committee due to the retrospective design of the study.

## Results

### Study population

**Fig 3:**
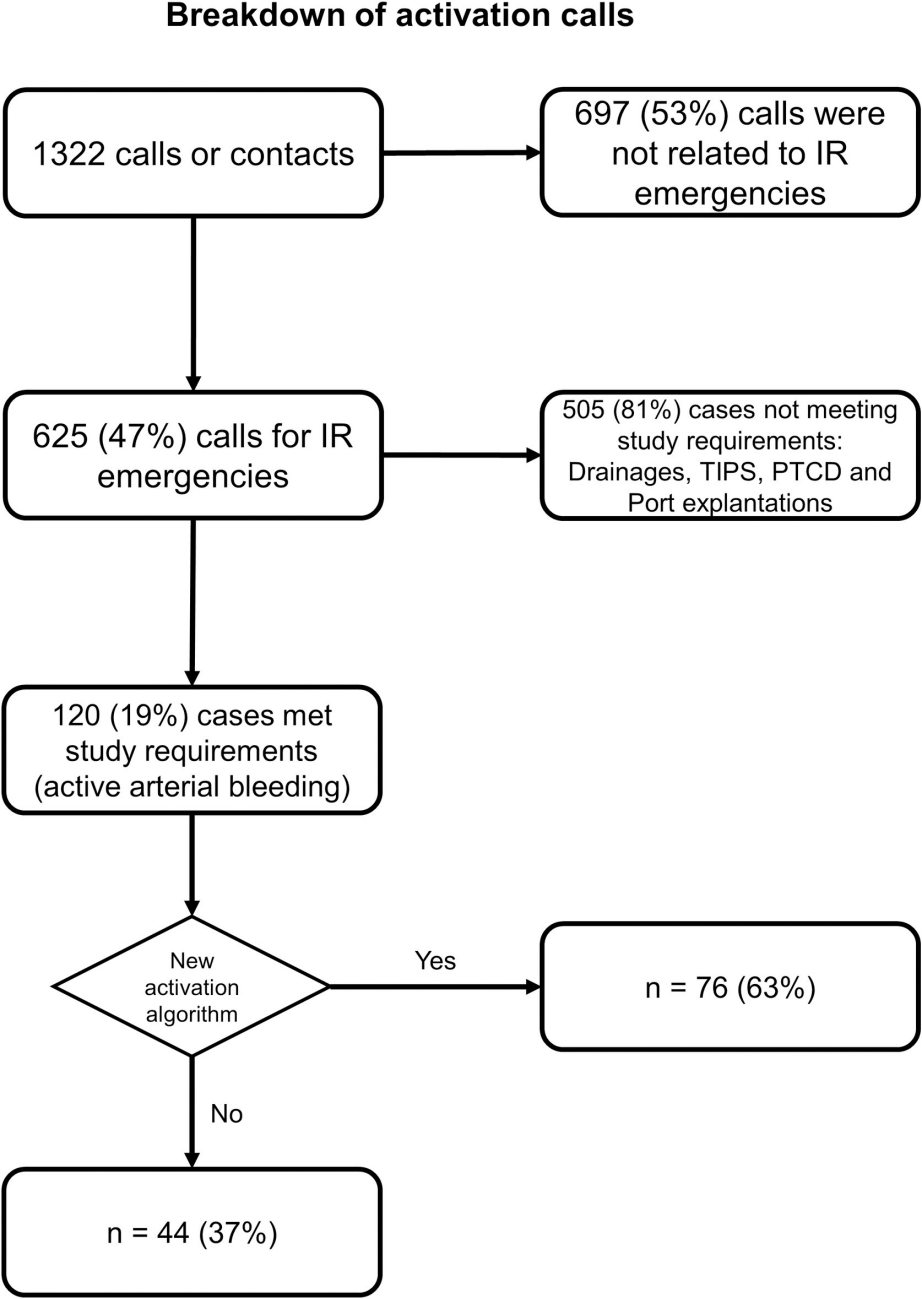
Illustrates the selection process for the study population.

During the study period, 1322 registered calls led to 625 emergency procedures, of which 120 bleeding interventions met the abovementioned requirements. In the study population, 44 patients (37%) were treated via the conventional activation pathway as opposed to 76 patients (63%), where contact was established using the emergency phone. In 49 cases (41%), treatment was performed during regular working hours compared with 71 cases (59%) outside regular working hours. Further details of the study population are provided in Table 1 and Fig 3.

**Table 1:** Patient characteristics and procedural data.

Characteristics (n = 120)	
Mean age in years	67.1 ± 14.8
Gender	63 male (53%)
Mean dose area product (cGy*cm2)	32212 ± 66250
Mean fluoroscopy time in min	23.6 ± 17.7
Bleeding vessel	
A. costalis	9 (8%)
A. femoralis profunda	12 (10%)
A. femoralis superficialis	11 (9%)
A. gastroduodenalis	11 (9%)
A. hepatica	9 (8%)
A. iliaca interna	8 (7%)
A. lienalis	8 (7%)
A. lumbalis	16 (13%)
A. mesenterica	5 (4%)
A. renalis	6 (5%)
A. subclavia	3 (3%)
V. femoralis	1 (1%)
Other vessel	21 (18%)
Type of vessel	
Arterial	103 (86%)
Venous	1 (1%)
Not detectable	16 (13%)
Main concept of treatment	
Detachable coils	12 (16%)
Detachable and pushable coils	1 (1%)
Stent graft	1 (1%)
Pushable coils	84 (76%)
Particles (e.g., polyvinyl alcohol)	4 (3%)
Spheres	2 (2%)
Technical success	104 (87%)
Mean interval from last CT image to first angiographic image in min	88.2 ± 46.3
Mean interval from last CT image to complete treatment in min	142 ± 53.4
Mean duration of intervention	52.5 ± 31.5
Cases outside regular working hours	0.592+-0.494
Activations via dedicated emergency phone	76 (63%)

This table presents key demographic and procedural data.

### Interventions

The treatment strategies, including the specification of bleeding vessels, are provided in Table 1. The technical success rate was 87%. In cases that were not considered technically successful, no bleeding was detectable at the time of intervention. No detectable bleeding remained untreated or insufficiently embolized. The activation algorithm utilizing the emergency phone led to a slight decrease in radiation doses and the fluoroscopy times by trend, as provided in Table 2.

**Table 2:** Comparison of interventional parameters: Use of emergency phone vs. non-use.

Interventional parameters in comparison			
	Without emergency phone	With emergency phone	p-value
Mean dose area product (cGy*cm2)	39450 ± 94444	27424 ± 37761	0.43
Mean fluoroscopy time in min	25.8 ± 22.7	22.1 ± 13.4	0.34
Technical success	30 (68.2)	71 (93.4)	***0*.*00049***
Interval: CT to treatment	103 ± 61.9	79.4 ± 31.6	***0*.*02***
Interval: CT to the end of treatment	159 ± 66.4	132 ± 41.6	***0*.*02***
Procedure time	56.7 ± 40.4	50.1 ± 25	0.34
Case outside regular working hours	26 (59.1%)	45 (59.2%)	0.99

This table compares interventional parameters between cases managed with and without the use of an emergency phone.

### Intervals and technical success

The mean interval between the last CT image and the first angiographic series was 88 ± 46 min, and the mean interval between the last CT image and the last angiographic series was 141 ± 53 min. In the mean, the intervention time was 45 ± 31 min. Before the implementation of the emergency phone mean interval between diagnosis and treatment was 95 ± 62min, and after the implementation of the emergency phone 80 ± 31min. The mean reduction of the diagnosis to treatment interval was 15 minutes and statistically significant (p = 0.019). A detailed description of intervals and parameters is provided in Table 2. After implementing the emergency phone, success rates increased significantly from 68% to 94% (p<0.001).

## Discussion

Interventional radiology often is confronted with acute and life-threatening conditions requiring immediate treatment. Besides the logistical challenges to the interventionalist and the patient, as well as other supporting specialties such as technicians and anaesthesiologists, effective communication structures are crucial [4, 6, 9, 11]. Nevertheless, publications about effective and standardized communication structures for activation algorithms in interventional radiology are unavailable. Even recent papers highlighting the importance of effective and quick communications underlines the need for more simplified structures [6, 9, 11, 12]. Although, the crucial role of phone call chains have been identified by several medical disciplines, data about the implementation of direct call routing has not been published yet [13]. In this study, we present the results after implementing a standardized activation pathway via an emergency phone that is constantly routed to a corresponding interventional radiologist and compare activation times before and after taking this measure.

The main findings of this study are i) the standardized activation pathway leads to shorter activation times allowing for faster treatment of patients after diagnosis of bleeding in CT. ii) implementation of the emergency phone leads to improved intervention quality. iii) improved communication structures for activation algorithms might lead to lower radiation exposure and procedure times.

On call physicians for after-hours compared to in-house physicians do not necessary delay treatment while the on-call concept is cost-efficient [14]. Activation of the interventional team often happens unstructured [6, 9]. That leads to inefficient communication pathways with parallel structures that may result in prolonged intervals before patients get lifesaving treatment with the inherent risk of additional information loss [1–3, 15]. The results of this study show a faster time to treatment and a lower standard deviation when activation was done via the emergency phone. This is an indicator of increased process quality. The activation via one IR emergency phone has several further advantages. First, clinical partners just need to remember one number. A simple number with repeated digits is advisable. Second, the simplified activation pathways facilitate information flow. Even when several specialties are responsible for the same patient, implementing the emergency phone makes the IR on-call the only recipient for all requests and requirements, omitting parallel communication. Thirdly, the emergency phone allows close contact between all participants and the department of interventional radiology. We experienced an increase in calls after the implementation of the phone and a slight amount of abuse. In some cases referring physicians call the emergency number so schedule urgent but elective interventions. Also when the regular line is occupied the emergency line is dialled for organisational purposes. However, the abuse is tolerable regarding the benefits of the improved activation algorithm. Fourthly, the emergency phone allows for more direct communication between the participants; the clinical partners can call the IR when they have patients with a clinical issue and discuss further steps. Then, in the following, the diagnostic radiologist can directly contact the IR when detecting the clinical cause or when unsure about the relevance of a finding. However, these aspects were outside the scope of the study.

In this study, we observed a highly significant increase in technical success rate after implementing the emergency phone. As stated under the result section, in all cases that lack technical success, no bleeding was detectable in the angio suite anymore. Considering that the emergency phone leads to faster reaction times of the IR with shorter intervals from diagnosis to treatment, the main reason for the higher success rate is probably the bleeding rates still being above the detection limit of digital subtraction angiography [16]. This is of note because non-detectable bleedings in the angio might still be hemodynamically relevant and even can increase bleeding rates again [16]. Although the procedure times after activation with the emergency phone were lower, the effect of a better-informed interventional radiologist because of improved communication structures is probably neglectable. Since introduction of the improved algorithm yearly phone contacts have multiplied; this probably partially linked to a more prominent appearance of IR in our clinic through the easy accessibility. The positive effects of the implementation of the new pathway were noticeable from the beginning which is why the improved activation algorithm is still in use.

### Future applications

At this point, the particular interventional radiologist on call does the emergency phone routing manually. Fully integrated systems that correspond with the duty roaster automatically could facilitate algorithms further. Failure concepts with automated re-routing of the calls in case of non-reachability of the corresponding IR could be used to introduce an additional layer of security. Artificial intelligence is also emerging as a powerful tool to reduce diagnosis to treatment time [8].

### Limitations

This is a single-centre retrospective study. The optimization is very individual for each clinic and for every department. The study’s findings might only be readily applicable to some other departments. In many cases, interventional radiology is closely associated with the diagnostic radiology department making IR particularly prone to ineffective activation processes. Radiation doses have a large devation; Body Mass Index(BMI) was retrospectively not available for the study population. However, the effect of BMI in our study is assumingly secondary because different anatomic structures have been treated such as legs and lower abdomens having a more relevant effect. Also oblique projections were used in some cases which also was not documented as precise dose evaluation is beyond the scope of this study. Treatments performed under general anaesthesia have not been assessed separately. Although general anaesthesia can prolong the diagnosis to treatment time; this effect has to be assumed to be equally distributed among both evaluated groups. The results of this study might differ for other departments like, e.g., surgery or gynaecology.

### Conclusion

Standardized and simplified communication algorithms can improve clinical workflow in emergencies in interventional radiology. The implementation of an emergency phone all-time routed to the mobile phone of the corresponding interventional radiologist on call leads to faster treatment of patients, higher procedure quality, and might also reduce radiation exposure.

## Supporting information

S1 Dataset Anonymized data set. (XLSX)

## References

[1] Gawande AA, Zinner MJ, Studdert DM, Brennan TA: Analysis of errors reported by surgeons at three teaching hospitals. *Surgery* 2003, 133(6):614–621. doi: 10.1067/msy.2003.169 12796727

[2] Guise J-M, Segel S: Teamwork in obstetric critical care. *Best practice & research Clinical obstetrics & gynaecology* 2008, 22(5):937–951. doi: 10.1016/j.bpobgyn.2008.06.010 18701352 PMC4987289

[3] Helmreich RL, Foushee HC: Why CRM? Empirical and theoretical bases of human factors training. In: *Crew Resource Management*. edn.: Elsevier; 2019: 3–52.

[4] Leonard M, Graham S, Bonacum D: The human factor: the critical importance of effective teamwork and communication in providing safe care. *BMJ Quality & Safety* 2004, 13(suppl 1):i85–i90. doi: 10.1136/qhc.13.suppl_1.i85 15465961 PMC1765783

[5] Reason J: Understanding adverse events: human factors. *BMJ Quality & Safety* 1995, 4(2):80–89. doi: 10.1136/qshc.4.2.80 10151618 PMC1055294

[6] Qazi E, Tao M, Oreopoulos G, Annamalai G, Mafeld S: Vascular Emergencies: When to Call an Interventional Radiologist. In: *Atlas of Emergency Imaging from Head-to-Toe*. edn.: Springer; 2022: 1–20.

[7] Loffroy R, Rao P, Ota S, De Lin M, Kwak B-K, Geschwind J-F: Embolization of acute nonvariceal upper gastrointestinal hemorrhage resistant to endoscopic treatment: results and predictors of recurrent bleeding. *Cardiovascular and interventional radiology* 2010, 33:1088–1100. doi: 10.1007/s00270-010-9829-7 20232200

[8] Katzman BD, van der Pol CB, Soyer P, Patlas MN: Artificial intelligence in emergency radiology: a review of applications and possibilities. *Diagnostic and Interventional Imaging* 2023, 104(1):6–10. doi: 10.1016/j.diii.2022.07.005 35933269

[9] Kim C, Niekamp A, Pillai AS, Leon R, Soni J, McNutt M, et al.: Quality Improvement Project: improving interventional radiology response times for Level I trauma embolization. *Journal of the American College of Radiology* 2020, 17(6):791–795. doi: 10.1016/j.jacr.2020.01.011 32068007

[10] Team RC: R: A Language and Environment for Statistical Computing. In., vol. R version 3.5.3 (2019-03-11) — "Great Truth"): R Foundation for Statistical Computing; 2019.

[11] Perera Molligoda Arachchige AS, Stomeo N: Rethinking Patient Communication in Radiology and Nuclear Medicine: Striking a Balance for Optimal Care. *Clinical and Translational Imaging* 2024, 12(2):225–228.

[12] Braun M, Schmidt WU, Möckel M, Römer M, Ploner CJ, Lindner T: Coma of unknown origin in the emergency department: implementation of an in-house management routine. *Scandinavian journal of trauma*, *resuscitation and emergency medicine* 2016, 24:1–8. 27121376 10.1186/s13049-016-0250-3 PMC4848793

[13] Lujak M, Billhardt H, Ossowski S: Optimizing emergency medical assistance coordination in after-hours urgent surgery patients. In: *Multi-Agent Systems*: *12th European Conference*, *EUMAS 2014*, *Prague*, *Czech Republic*, *December 18–19*, *2014*, *Revised Selected Papers 12*: 2015: Springer; 2015: 316–331.

[14] Demarest GB, Scannell G, Sanchez K, Dziwulski A, Qualls C, Schermer CR, et al.: In-house versus on-call attending trauma surgeons at comparable level I trauma centers: a prospective study. *Journal of Trauma and Acute Care Surgery* 1999, 46(4):535–542. 10.1097/00005373-199904000-00001 10217215

[15] Krug SE: The art of communication: strategies to improve efficiency, quality of care and patient safety in the emergency department. *Pediatric radiology* 2008, 38(Suppl 4):655–659. doi: 10.1007/s00247-008-0893-y 18810415

[16] Hermie L, Dhondt E, Vanlangenhove P, De Waele J, Degroote H, Defreyne L: Empiric cone-beam CT-guided embolization in acute lower gastrointestinal bleeding. *European Radiology* 2021, 31:2161–2172. doi: 10.1007/s00330-020-07232-7 32964336

